# Radiology curriculum

**Published:** 2008-11

**Authors:** KP Mody

**Affiliations:** Department of Radiology, Tata Memorial Hospital, Mumbai, India IJR, Vol. V. May 1951, No. 1

## “Ourselves”

Owing to circumstances beyond our control, the publication of our journal had to be discontinued for several months. This has been due to the very regrettable action of the Madras Government prohibiting its officer from holding honorary appointments in any Scientific organisation. As a result of this ban our General Secretary and or Editor-in-Chief had to resign all of a sudden before suitable substitutes could be appointed. All our requests for re-consideration were turned down even though it was pointed out that our activities would receive a serve set back. The publication of the Journal had to be transferred from Madras to Bombay, where it has settled down after months of anxiety and uncertainty. Government's action preventing their officers from holding such appointment as entail the handling of Society funds, though not justifiable, is perhaps understandable. But the post of Editor of a Scientific Journal carries no such responsibilities and there was no valid reason for preventing one of their officers from such activity. The functions of an Editor are purely academic and Government should encourage such activities rather than stop their officers from performing duties which further and foster the cause of science. We hope the Government will soon see their way to rescind the order.

One of the most important activities of our Association is the publication of the journal. The first issue of the journal came out in February 1947. Dr K. Manjunath Rai and Dr. Rama Rau were the Joint Editors, and Dr U. Krishna Rau of the “Antiseptic” kindly agreed to print it in his “Antiseptic” press. In 1947 a new Editorial Board was formed with Dr. K Manjunath Rai as the Editor in Chief. He was assisted by four members. The journal has been well received both in India and aboard. We earnestly appeal to our members to contribute freely to its columns. It is all well and good that we meet once a year and discuss various problems and read papers. Many members think that if they attend our annual conference they have done their duty. That is not so. When we part, continuity is lost and we return to our own small world in professional life with its narrow and mercenary interests. Contributions to the journal keep up continuity of our annual contacts, and keep alive scientific interest in our daily routine. For those who are fortunate enough to be attached to the Hospitals, scientific should come easy with a little extra effort. There is always plenty of material, all that is required is a proper sifting to give it a scientific bent. But even for a large majority who have no such opportunities and facilities as is obtainable in hospitals, even for these their daily routine must furnish them with opportunities of bringing out their problems and discussing them in the pages of the journal. Such contribution could be interesting and instructive. We feel certain this appeal will evoke a suitable response.

The year 1950 was a memorable year. The 6^th^ international Congress of Radiology was held in London in July. It was attended by Delegates form all over the world. Over 3000 delegates and associates attended the Congress, which was a great success. India had the honour of being invited to send a Delegation. India had a seat on the Executive Committee and moreover the Chairman of the Delegation was appointed as a member of the International Committee for Staging and Presentation of results of Treatment. Dr K. P. Mody gave a talk on his visit and his impression of the Congress to the meeting recently held in Calcutta. He gave a short resume of the Congress activities. Due to the co-operation and collaboration of the Radiologists and Physicists of Great Britain, the congress was of great success. In this stupendous undertaking our British colleagues have set up an example to the world, how smoothly and successfully a carefully planned programme could be put together.

The 5^th^ All Indian Congress of Radiology was held in Calcutta in February this year. Captain M. B. Mukherji was unanimously elected as President. No better choice could have been made. Apart from his high professional attainments and reputation. He is a well know figure in Calcutta with a bold independent outlook. The Presidential address was delivered before a large and influential gathering, on the inauguration day. He touched on all aspect of our speciality, our difficulties and our shortcomings, our achievements and aspirations. Many of his observation command attention.

The inaugural address was delivered by Dr. D M Bose the distinguished scientist, Director of the Bose Institute, an Institution founded by his uncle the late Sir Jagadish, world renowned for his scientific achievements. This was 2^nd^ time we had the privileges of listening to his address.

Many important problems were discussed, pertaining to the practice of Radiology in India. Owing to the import license policy of the Government of India adequate supply of films in India may be jeopardised. It has not been sufficiently well realised that there is a great demand for films in Europe, from were we draw our supplies. If we have to be assured of a steady supply of films order have to be placed months ahead so that the quota may be reserved for India. It is true that the imports of films are put on an open general licence. But unfortunately this holds good on for a limited period of time. There is always uncertainty of its not being renewed at the expiry of the period, so that local agents of films manufacturers are not in a position to place our requirements with their home factories well in advance. May we suggest to Government that the open license be made applicable for a reasonable period, say three years?

The supply of Chemicals is in a deplorable stage. It is not on open general license and supplies are short. An enterprising firm is manufacturing these locally but their output is totally in adequate. It is imperative that the chemicals should also be placed on an open license, at any rate till such a time as Indian manufacturers can cope with the demand. What good are films without chemicals? Moreover they should be of the best possible quality.

Another anomalous position is that of the import of X-rays instruments and accessories. Instruments can be imported without a license but not so the accessories such as screens, cassettes, hangers etc. These require import permits a very untenable, position, which ought to be rectified immediately.

The question of manufactures of x-ray machine has been engaging our attention for the last several years. In view of the absence of basic electrical industries in the country, it is a moot question whether we could manufacture such specialised machinery on an economical basis. The demand for such machinery is after all limited, the industrialist in any country would like to be assured of reasonable profit and a stable market before launching into a scheme for the manufacture of highly technical and specialised machines. The project requires careful planning and great deal of thought before such an industry can be profitable launched.

No problem is a greater importance to the future of our profession than that of imparting sound education to our post-graduates so that they may be fully qualified to practice the speciality of Radiology. At present sporadic attempts have been made to impart some sort of specialised education, diploma courses have been established in Bombay, Madras and Lucknow, for the last few years. Lasting as they do for 9 years to one year it is easy to understand that the standard falls far short of that prevailing in the United Kingdom and America. Our energetic General Secretary brought this important subject up before the General Body and sponsored a resolution to go into the entire question thoroughly. Three sub-committees have been formed, in Bombay, Madras, and Calcutta and we shall await their reports before commenting further on this important subject.

A subject of paramount importance to Radiologists is the question as to who should be permitted to practice the speciality. It is only too well known that many medical men start practising as “radiologists”, without any special knowledge of the subject, simply because they have been able to invest in the purchase of the X-ray machine. The Government of India in 1948 asked for our opinion on the matter, as they were contemplating “legislative and administrative action to control appropriately the use of radiation for therapeutic and diagnostic purpose.” This question was referred to several members who have already expressed their opinion on the subject. The matter was again referred to the Central Council meeting held in Calcutta this year and it was resolved to go into the question thoroughly and send the recommendation to the Central Government. As regards the use of radiation in Therapy, there are no two opinions, only those who hold certificate of adequate training in Therapy department should be permitted to practice. But as regards Radio-diagnosis, the question is complicated by the fact that many Physicians and Surgeons posses X-ray plant for assisting them in diagnosing their own cases. Since many of these medical men hold high qualifications, it is moot point how far and to what extend should legislation affect them in the practice of their routine activities which involves the use of X-rays.

